# Withdrawing guideline-directed medical therapy after left ventricular ejection fraction recovery following atrial fibrillation ablation: a multicentre cohort study

**DOI:** 10.1136/openhrt-2025-003733

**Published:** 2025-10-13

**Authors:** Sayed Al-Aidarous, Saffron Rajappan, Nikhil Ahluwalia, Christopher P Uy, Hatem Abdelgawad, Caterina Vidal Horrach, Sofiane Kouadria, Zhen Hua, Gurkiran Sandhar, Theo Cooke, Salman Rasheed, Suria Geran, Kayla Li Xian Chiew, Meher Lehri, Dimitrios Palaiologos, Arsalan Khalil, Brett Kennedy, Richard Balasubramaniam, Shahana Hussain, Lauren Stanton, Syed Ahsan, Christopher Primus, Anthony W C Chow, Martin Thomas, Amal Muthumala, Syed M A Sohaib, Richard Ang, Nikolaos Papageorgiou, Charles Butcher, Kim Rajappan, Caroline Roney, Ross J Hunter, Shohreh Honarbakhsh

**Affiliations:** 1 St Bartholomew’s Hospital, London, UK; 2 Queen Mary University of London, London, UK; 3 UCLMS, University College London Medical School, London, UK; 4 Cardiac Department, Oxford University Hospitals NHS Trust, Oxford, UK; 5 Barking Havering and Redbridge Hospitals NHS Trust, Romford, UK; 6 Royal Bournemouth Hospital, University Hospitals Dorset NHS Foundation Trust, Poole, UK; 7 Lister Hospital, Stevenage, UK; 8 Cardiology, Whittington Hospital, London, UK; 9 Imperial College London National Heart and Lung Institute, London, UK; 10 Homerton University Hospital NHS Trust, London, UK; 11 Cardiology, North Middlesex University Hospital, London, UK; 12 Royal Berkshire Hospital, Reading, UK; 13 Dorset County Hospital NHS Foundation Trust, Dorchester, UK; 14 University College London Hospitals NHS Foundation Trust, London, UK; 15 University Hospitals Dorset NHS Foundation Trust, Poole, UK

**Keywords:** Atrial Fibrillation, Heart Failure, Systolic, HEART FAILURE, Cardiac Catheterization

## Abstract

**Background:**

Atrial fibrillation (AF)-induced cardiomyopathy (AIC) is characterised by reversible left ventricular (LV) dysfunction after restoration of sinus rhythm (SR). The need for continued guideline-directed medical therapy (GDMT) for heart failure after LV ejection fraction (LVEF) recovery in AIC after catheter ablation (CA) is unclear.

**Methods:**

This multicentre cohort study across 12 UK centres included adults undergoing index AF ablation (June 2019–June 2024) with LVEF <50% preablation and recovery to ≥50% at three timepoints: preablation; early postablation (≥4 weeks) and late postablation (≥3 months or ≥3 months post-GDMT withdrawal). Patients were stratified post recovery of LVEF after CA. The primary outcome was mean LVEF at late follow-up; secondary outcomes included absolute change in LVEF, LV end-diastolic diameter (LVEDD) and SR maintenance.

**Results:**

88 patients met inclusion enrolment criteria (61.7±10.6 years old; 91% male), of which 50 (56.8%) continued full-dose GDMT and 38 (43.2%) withdrew ≥50% of GDMT. In the GDMT-withdrawn group, mean GDMT classes decreased from 2.97±0.88 to 1.03±0.79 (p<0.001). At late follow-up, mean LVEF was comparable (56.3%±3.8% GDMT-continued vs 56.8%±5.5% GDMT-withdrawn; p=0.59), as was LVEF change (1.2% vs 0.4%; p=0.48). One relapse occurred in each group secondary to an acute coronary syndrome (2.0% vs 2.6%; p=1). LVEDD remained stable (p>0.8). SR was maintained in 82.0% vs 92.1% of patients; p=0.17.

**Conclusions:**

Selective GDMT withdrawal after sustained LVEF recovery and rhythm control did not compromise LV systolic function, remodelling or rhythm maintenance. This supports the study of personalised de-escalation strategies in AIC in prospective trials.

**WHAT IS ALREADY KNOWN ON THIS TOPIC**

- Atrial fibrillation-induced cardiomyopathy (AIC) can reverse with restoration of durable sinus rhythm (SR) after catheter ablation, but current guidelines mandate lifelong guideline-directed medical therapy (GDMT) despite risks and burdens. Prior trials in non-ischaemic cardiomyopathy and heterogeneous heart failure with reduced ejection fraction cohorts have shown high relapse rates of left ventricular (LV) dysfunction on GDMT withdrawal, leaving uncertainty for the AIC post catheter ablation population.

**WHAT THIS STUDY ADDS**

- This multicentre study including patients with AIC with recovered LV ejection fraction (LVEF) following restoration of SR after catheter ablation has shown that selective withdrawal of ≥50% of GDMT classes results in no relapse of LV dysfunction with preserved LVEF, stable ventricular dimensions and durable SR maintenance when compared with patients who continued full-dose therapy over a 32-month follow-up.

**HOW THIS STUDY MIGHT AFFECT RESEARCH, PRACTICE OR POLICY**

- These findings support the feasibility of personalised GDMT de-escalation in AIC and justify prospective, randomised trials to define which patients can safely discontinue chronic heart-failure therapies, potentially reducing medication burden and healthcare costs without compromising outcomes.

## Introduction

 Arrhythmia-induced cardiomyopathy is a distinct, reversible form of left ventricular (LV) dysfunction driven by sustained tachyarrhythmias, most commonly atrial fibrillation (AF).^1^ Persistent, high-rate and irregular ventricular activation during AF provokes intracellular calcium dysregulation,^2^ oxidative stress^3^ and ventricular fibrosis^4^ leading to adverse LV remodelling and systolic impairment.^5^ Crucially, restoration of sustained sinus rhythm (SR), most effectively accomplished by catheter ablation, can yield marked recovery of LV ejection fraction (LVEF), reverse remodelling and symptomatic relief.^6^,^8^

Contemporary management of AF-induced cardiomyopathy (AIC) couples control of the arrhythmia with guideline-directed medical therapy (GDMT) for heart failure with reduced ejection fraction (HFrEF). Although GDMT reduces mortality and hospitalisation in HFrEF, the evidence is largely derived from patients with ischaemic and non-ischaemic cardiomyopathies and its continued role after LVEF normalisation in patients with AIC is untested. Furthermore, GDMT imposes substantial burdens including annual drug costs of ~US$10 000,^9^ mandatory laboratory surveillance^10^ and risks of hypotension,^11^ bradycardia,^12^ renal impairment^13^ and electrolyte disturbances.^14^ Trials of GDMT withdrawal in mixed HFrEF cohorts report relapse rates of up to 40%, whereas distinct reversible syndromes have shown relapse rates of <10%.^15^ The ‘withdrawal of pharmacological treatment for HF in patients with recovered dilated cardiomyopathy’ (TRED-HF) study evaluated HF GDMT withdrawal in patients with idiopathic DCM who recovered LV systolic function following GDMT initiation.^16^ Participants were randomised to withdrawal or continuing pharmacological HF treatment. The primary endpoint was a relapse of DCM within 6 months, defined by reduction in LVEF of more than 10% and to less than 50%, an increase in LV end-diastolic volume by more than 10% and to higher-than-normal range, a twofold rise in N-terminal-pro-B-type natriuretic peptide concentration and to more than 400 ng/L, or clinical evidence of HF. Over the first 6 months, 44% of patients randomly assigned to treatment withdrawal met the primary endpoint of relapse compared with none of the patients assigned to continued treatment. The conclusion from this study was that many patients who have recovered from DCM will relapse following treatment withdrawal, thereby recommending continuation of treatment. This study focused on patients with DCM, of which a majority had no clear reversible cause and no patients had AIC. It is plausible that in these patients the recovery seen in the LV systolic function was secondary to the pharmacological HF treatment and thus, withdrawal of this resulted in relapse of the LV systolic dysfunction (LVSD). However, patients diagnosed with AIC, who demonstrate normalisation of LVEF following treatment of the inherently culpable arrhythmia may not require continued HF pharmacological therapy to retain a normal LVEF.^17^ Thereby, it remains unclear whether patients with a reversible cause for their LVSD, such as with AIC, would have the same response to the withdrawal of pharmacological HF treatment.

The aim of this multicentre retrospective observational cohort study that used prospectively collated data from 12 UK centres was to evaluate the safety of withdrawing GDMT in patients with AIC whose LV function has recovered in SR after AF catheter ablation. It was hypothesised that, in the setting of a normalised LVEF postcatheter ablation, targeted GDMT de-escalation would not precipitate LVSD relapse.

## Methods

A multicentre retrospective observational cohort study that used prospectively collated data from twelve UK centres to evaluate the safety of de-escalating GDMT after LVEF recovery following restoration of SR with catheter ablation in patients with AIC. These centres were Barts Heart Centre, Dorset County Hospital, Homerton Hospital, John Radcliffe Hospital, King George Hospital, Lister Hospital, North Middlesex Hospital, Queen’s Hospital, Royal Berkshire Hospital, Royal Bournemouth Hospital, University College London Hospital and Whittington Hospital.

Institutional catheter ablation databases were reviewed to identify all patients undergoing a first catheter ablation for AF between June 2019 and June 2024.

From this pool, patients meeting the following criteria were included:

- Preablation LVSD with LVEF <50%.
- Recovery of LV systolic function following restoration of SR with catheter ablation, defined as an LVEF ≥50% and at least a 5% increase in LVEF from preablation on any follow-up imaging modality.
- Imaging at three time points:
  - A—preablation.
  - B—Early postablation: first imaging ≥4 weeks postablation. Where patients underwent more than one procedure, this was defined as the first echocardiogram that was performed ≥4 weeks after the final ablation.
  - C—Late post ablation—≥3 months after early post ablation imaging—or, if GDMT was withdrawn, ≥3 months after cessation.

Patients with congenital heart disease or missing imaging/medication data at the three key time points were excluded (online supplemental figure 1). No exclusions were made based on New York Heart Association (NYHA) class, presence of implantable devices, other comorbidities, late gadolinium enhancement (LGE) on cardiac MR imaging (CMR) or number of catheter ablations. Individual consent was not required due to the anonymised retrospective nature of the study. This was a retrospective analysis of routinely collected clinical data; no patients or members of the public were involved in its design or conduct.

### Endpoints and outcome measures

#### Primary outcome

LVEF at time point C (late postablation) compared between patients who continued GDMT and those who withdrew ≥50% of GDMT classes.

#### Secondary outcomes

- Absolute change in LVEF from timepoint B to C compared between patients who continued GDMT and those who withdrew ≥50% of GDMT classes.
- Proportion of patients with recurrent LVSD late postablation (timepoint C) defined as an LVEF <50%.
- Arrhythmia recurrence comprising any documented AF or atrial tachycardia lasting >30 s off anti-arrhythmic drugs on ECG, Holter or device interrogation after the 3-month blanking period.^18^

### Data collection

Baseline demographic data, clinical and imaging variables were extracted from electronic records. GDMT classes were recorded including ß-blockers, ACE inhibitors (ACEi)/angiotensin II receptor blockers (ARBs)/angiotensin receptor-neprilysin inhibitors (ARNI), mineralocorticoid receptor antagonists (MRA) and sodium-glucose cotransporter-2 (SGLT2) inhibitors. Data were reviewed to ensure patients were on optimal doses of GDMT classes as tolerated by their blood pressure and heart rate.

### AF ablation

AF catheter ablations were performed under conscious sedation or general anaesthesia using either radiofrequency energy or cryoablation per the institutional protocol.

### Imaging and LVEF assessment

All transthoracic echocardiography (TTE) adhered to the British Society of Echocardiography guidelines with LVEF calculated by Simpson’s biplane method.^19^ LV end-diastolic diameter (LVEDD) was measured inner edge to inner edge, perpendicular to the long axis of the LV at end-diastole defined as the frame with the largest LV dimension.^20^ The LA diameter was measured on the parasternal long-axis view from the posterior aortic wall to the posterior LA wall at the end-ventricular systole just before the mitral valve opening.^20^ LA volume was measured using the modified biplane area-length method using the 2-chamber and 4-chamber views and was corrected for body surface area.^20^ When available, CMR LGE was noted. LVEF recovery was defined as ≥50% on the early post-ablation TTE (time point B).

### HF therapy de-escalation

Decisions to continue or withdraw GDMT were at the treating physician’s discretion without prespecified criteria or taper protocol. Withdrawal was defined as cessation of ≥50% of GDMT classes; continuation required maintenance of all initial HF agents.

### Follow-up and rhythm surveillance

Patients were reviewed clinically 3 months postablation and thereafter at variable intervals in outpatient clinics. Rhythm monitoring, including Holter monitors, symptom-triggered ECGs or device interrogations, was performed per routine care; data from implantable loop recorders or cardiac implantable electronic devices were used when present. Maintenance of SR required absence of AF/atrial tachycardia >30 s on any available recording through final follow-up.

### Statistical analysis

All analyses were conducted using IBM SPSS Statistics, V.29. The distribution of continuous variables was assessed with the Shapiro-Wilk test. Parametric data are presented as mean±SD and compared using a Student’s t-test (two groups). Non-parametric data are reported as median (IQR) and compared using the Mann-Whitney U test (two groups) or Fisher’s exact test. Categorical variables are expressed as counts (percentages) and compared with the χ² test or Fisher’s exact test, as appropriate. A p<0.05 was deemed significant.

## Results

**Figure 1:**
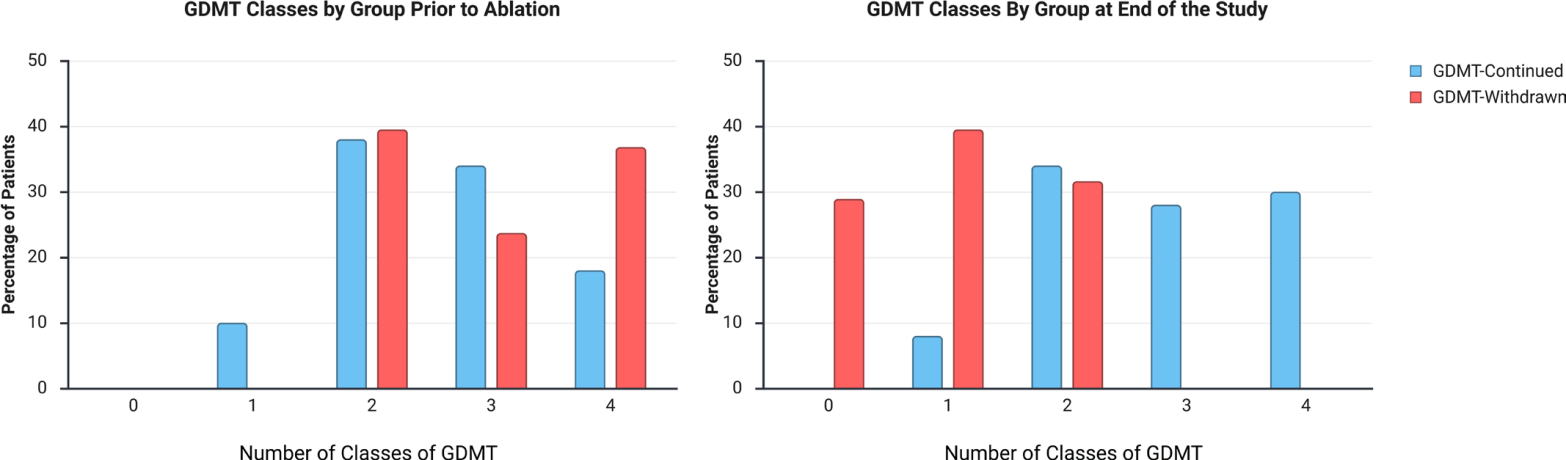
Demonstrates the GDMT classes in the GDMT-continued and GDMT-withdrawn groups prior to ablation and late post ablation. The figure demonstrates that postablation, the patients in the GDMT-continued group remained on their GDMT classes, while in the GDMT-withdrawn group, a large proportion of patients withdrew all their medication, with no patients remaining on three or four GDMT classes. GDMT, guideline-directed medical therapy.

A total of 3803 patients underwent index AF ablation between June 2019 and June 2024 across the 12 centres. After applying inclusion and exclusion criteria (figure 1), 88 patients with AIC and complete follow-up data were analysed: 50 (56.8%) continued full-dose GDMT and 38 (43.2%) withdrew ≥50% of GDMT classes. Mean postablation follow-up was 32.0±21.5 months overall, 34.9±22.3 months in the continued group vs 27.7±19.8 months in the GDMT-withdrawn group (p=0.15). Of the patients who met the study criteria, 5 (5.7%) patients increased their LVEF by 5%–10% and the remaining 83 (94.3%) increased their EF by >10%. Echocardiograms were done in SR.

### Baseline characteristics

88 patients were included (mean age 61.7±10.6 years; 90.9% male; mean BMI 30.1±4.8 kg/m²). Common comorbidities were hypertension (43.2%), diabetes (14.8%) and prior cerebrovascular attack/transient ischaemic attack (11.4%). The mean number of AF ablations per patient was 1.5±0.7. Out of the 88 patients, 53 (60.2%) patients had one AF ablation, 28 (31.8%) had two AF ablations, 4 (4.5%) had three AF ablations and 3 (3.4%) had four AF ablations. The majority of patients had persistent AF at baseline (n=78, 88.6%). When stratified by GDMT strategy, the two groups were well matched for demographics, body habitus, AF subtype, comorbidities and number of ablations (table 1). There was no significant difference in age between the two groups (61.7±9.6 years GDMT-continued vs 61.7±12.0 years GDMT-withdrawn; p=0.99). There was also no significant difference in the proportion of patients with persistent AF (n=45, 90.0% GDMT-continued vs n=33, 86.8% GDMT-withdrawn; p=0.74) or paroxysmal AF (n=5, 10% GDMT-continued vs n=5, 13.2% GDMT-withdrawn; p=0.74) across the two groups. The number of ablations was similar between the GDMT-continued (1.6±0.8) and GDMT-withdrawn (1.4±0.7) groups (p=0.32). All patients had antiarrhythmic drugs stopped at 3 months postablation.

**Table 1:** Baseline demographic characteristics and comorbidities combined across the whole study cohort and subsequently across the two groups; those who have continued GDMT and those who have withdrawn GDMT

Demographics and comorbidities	Whole cohort (n=88)	GDMT-continued (n=50)	GDMT-withdrawn (n=38)	P value
Age years, mean±SD	61.7±10.6	61.7±9.6	61.7±12.0	0.99
Gender Male, n (%)	80 (90.9)	46 (92.0)	34 (89.5)	0.72
BMI kg/m², mean±SD	30.1±4.8	30.6±5.2	29.6±4.4	0.34
Hypertension, n (%)	38 (43.2)	21 (42.0)	17 (44.7)	0.78
Diabetes, n (%)	13 (14.8)	9 (18.0)	4 (10.5)	0.38
Ischaemic heart disease, n (%)	6 (6.8)	5 (10.0)	1 (2.6)	0.23
Cardiac surgery, n (%)	1 (1.1)	1 (2.0)	0 (0)	1.00
CVA/TIA, n (%)	10 (11.4)	4 (8.0)	6 (15.8)	0.32
COPD, n (%)	2 (2.3)	1 (2.0)	1 (2.6)	1.00
Obstructive sleep apnoea, n (%)	4 (4.5)	1 (2.0)	3 (7.9)	0.31
AF type—persistent, n (%)	78 (88.6)	45 (90.0)	33 (86.8)	0.74
AF type—paroxysmal, n (%)	10 (11.4)	5 (10.0)	5 (13.2)	0.74
Pre-ablation CMR performed, n (%)	56 (63.6)	32 (64.0)	24 (63.2)	0.94
Presence of LGE, n (%)	27 (48.2)	17 (53.1)	10 (41.7)	0.4
Total number of ablations mean±SD	1.5±0.7	1.6±0.8	1.4±0.7	0.32
One ablation, n (%)	53 (60.2)	27 (54.0)	26 (68.4)	0.09
Two ablations, n (%)	28 (31.8)	19 (38.0)	9 (23.7)	0.35
Three ablations, n (%)	4 (4.5)	2 (4.0)	2 (5.3)	1.00
Four ablations, n (%)	3 (3.4)	2 (4.0)	1 (2.6)	1.00
QRS Duration ms, mean±SD	101.5±20.4	99.8±16.6	103.6±24.5	0.45
Presence of LBBB, n (%)	2 (2.3)	1 (2.0)	1 (2.6)	1.00

AF, atrial fibrillation; BMI, body mass index; CMR, cardiac MR; COPD, chronic obstructive pulmonary disease; CVA/TIA, cerebrovascular attack/transient ischaemic attack; GDMT, guideline directed medical therapy; LBBB, left bundle branch block; LGE, late gadolinium enhancement.

### Baseline ECG, echocardiographic and CMR parameters

All patients had their preablation echocardiogram performed in AF and timepoint B echocardiogram performed in SR. In the 88 patients making up the whole cohort, the average LVEF and LVEDD was 35.2%±8.9% and 53.8±6.8mm, respectively. Out of these patients, 2 (2.3%) patients had underlying left bundle branch block. A cardiac MRI had been performed in 56 patients, in whom 27 (48.2%) had underlying LGE. As shown in table 2, baseline QRS duration, LV dimensions and left atrial size did not differ between groups. The proportion of patients with underlying left bundle branch block at baseline was not significantly different in the two groups (n=1, 2.0% GDMT-continued vs n=1, 2.6% GDMT-withdrawn; p=1.00).

**Table 2:** Echocardiographic parameters across all patients in the study and subsequently across the two groups; those who have continued GDMT and those withdrawn from GDMT

Parameter	Pre ablation (time point A)				Early post ablation (time point B)			
	Whole cohort	GDMT- continued (n=50)	GDMT- withdrawn (n=38)	P value	All patients	GDMT- continued (n=50)	GDMT- withdrawn (n=38)	P value
LVEF (%, mean±SD)	35.2±8.9	35.8±9.5	34.4±8.0	0.45	55.7±3.8	55.1±3.4	56.4±4.2	0.10
LVEDD (mm, mean±SD)	53.8±6.8	53.8±7.1	53.6±6.4	0.90	52.9±8.0	52.9±5.5	52.8±10.7	0.93
LA diameter (mm, mean±SD)	42.5±9.9	42.2±11.7	43.0±7.0	0.80	37.8±11.5	40.3±12.1	34.5±10.0	0.07
LA volume index (mL/m², mean±SD)	47.3±14.2	50.1±13.3	43.6±15.0	0.09	36.6±14.7	39.2±12.6	32.7±16.4	0.10

GDMT, guideline directed medical therapy; LA, left atrium; LVEDD, left ventricular end diastolic diameter; LVEF, left ventricular ejection fraction.

Mean preablation LVEF was 35.2%±8.9% in the whole cohort, 35.8%±9.5% in the GDMT-continued group (n=50) and 34.4%±8.0% in the GDMT-withdrawn group (p=0.45). The proportion of patients who underwent CMR in the groups was not significantly different (n=32, 64.0% GDMT-continued vs n=24, 63.2% GDMT-withdrawn; p=0.94) and there was no significant difference in the proportion of patients who had underlying LGE on CMR (n=17, 53.1% GDMT-continued vs n=10, 41.7% GDMT-withdrawn; p=0.40). Additionally, there was no difference in LVEF at postablation-TTE (time point B) between the GDMT-continued and the GDMT-withdrawn groups (55.1%±3.4% GDMT-continued vs 56.4%±4.2% GDMT-withdrawn; p=0.10).

### GDMT at baseline

At the time of catheter ablation, 86 patients (97.7%) were taking β-blockers, 82 (93.2%) ACE-inhibitors/ARBs/ARNIs, 49 (55.7%) MRAs and 26 (29.5%) SGLT2 inhibitors (table 3). There were no significant differences in baseline GDMT use between groups (table 3). In the whole cohort, the average number of GDMT classes at baseline was 2.8±0.91. The average number of GDMT classes at baseline was not significantly different between the GDMT-continued group (2.60±0.90) vs GDMT-withdrawn group (2.97±0.88; p=0.10). The proportion of patients taking 4 GDMT classes at baseline was not significantly different between the two groups (n=9, 18.0% GDMT-continued vs n=14, 36.8% GDMT-withdrawn; p=0.08).

**Table 3:** The heart failure medication taken at baseline across the whole cohort and subsequently categorised by GDMT status

Medications at enrolment	Whole cohort (n=88)	GDMT-continued (n=50)	GDMT-withdrawn (n=38)	P value
ACE inhibitor or ARB or ARNI, n (%)	82 (93.2)	45 (90.0)	37 (97.4)	0.23
ß blocker, n (%)	86 (97.7)	48 (96.0)	38 (100.0)	0.50
MRA, n (%)	49 (55.7)	26 (52.0)	23 (60.5)	0.43
SGLT2 inhibitor, n (%)	26 (29.5)	11 (22.0)	15 (39.5)	0.08
Anticoagulation, n (%)	88 (100)	50 (100)	38 (100)	–

ARB, angiotensin receptor blocker; ARNI, angiotensin receptor-neprilysin; GDMT, guideline-directed medical therapy; MRA, mineralocorticoid receptor antagonist; SGLT2, sodium-glucose cotransporter-2.

### Medication changes at final follow-up

By the end of follow-up, the GDMT-withdrawn group had substantially reduced use of all GDMT classes (table 4). All patients in the GDMT-withdrawn group withdrew more than 50% of GDMT classes, and no patients were excluded based on the number of GDMT classes withdrawn. No patients withdrew GDMT classes due to issues with tolerance, and no patients required reinitiation of any GDMT class after withdrawal during follow-up. In the GDMT-continued group, no patients had a reduction in their GDMT dosage during follow-up.

**Table 4:** The medications taken at the final follow-up across the whole cohort and subsequently categorised by GDMT status

Medications at final follow-up	Whole cohort (n=88)	GDMT-continued (n=50)	GDMT-withdrawn (n=38)	P value
ACE inhibitor or ARB or ARNI, n (%)	55 (62.5)	45 (90.0)	10 (26.3)	<0.001
ß blocker, n (%)	71 (80.7)	48 (96.0)	23 (60.5)	<0.001
MRA, n (%)	27 (30.7)	26 (52.0)	1 (2.6)	<0.001
SGLT2 inhibitor, n (%)	25 (28.4)	20 (40.0)	5 (13.2)	0.005
Anticoagulation, n (%)	75 (85.2)	47 (94.0)	28 (73.7)	0.009

ARB, angiotensin receptor blocker; ARNI, angiotensin receptor-neprilysin; GDMT, guideline-directed medical therapy; MRA, mineralocorticoid receptor antagonist; SGLT-2, sodium-glucose cotransporter-2.

The proportion of patients taking four GDMT classes was significantly different between the two groups (n=15, 30.0% GDMT continued vs n=0, 0% GDMT withdrawn; p<0.001). Compared with patients in the GDMT-continued group, patients in the GDMT-withdrawn group were significantly less likely to be taking:

- ACE-inhibitor/ARB/ARNI: 90.0% vs 26.3% (p<0.001).
- β-blocker: 96.0% vs 60.5% (p<0.001).
- MRA: 52.0% vs 2.6% (p<0.001).
- SGLT2 inhibitor: 40.0% vs 13.2% (p=0.005).

Anticoagulation use was also significantly less in the GDMT-withdrawn group compared with the GDMT-continued group at final follow-up (n=28, (73.7%) GDMT-withdrawn vs n=47, (94.0%) GDMT-continued; p=0.009). Additionally, no patients continued antiarrhythmic drugs beyond the 3-month blanking period, with the exception of beta blockers as reported above.

### Classes of optimal dose of GDMT

The mean number of GDMT classes per patient fell from 2.97±0.88 at baseline to 1.03±0.79 at final follow-up in the GDMT-withdrawn group (p<0.001), whereas it remained stable in the GDMT-continued group (2.60±0.90 to 2.78±0.97; p=0.11). The number of classes of GDMT at the end of follow-up was significantly lower in the GDMT-withdrawn group (1.03±0.79 GDMT-withdrawn vs 2.78±0.97 GDMT-continued; p<0.001) (figure 1 and online supplemental figure 2).

The distribution of patients by number of GDMT classes illustrates that by study end, 28.9% of the GDMT-withdrawn group were off all therapies compared with none in the continued group (table 5). In the GDMT-withdrawn group, no patients remained on three or four GDMT classes, while in the GDMT-continued group, 13 (26.0%) and 15 (30.0%) remained on three and four GDMT classes, respectively. In the GDMT-withdrawn group, a majority only remained on one GDMT class (n=15, 39.5%), of which this was most commonly a ß-blocker (n=12, 80%). In an exploratory subgroup analysis of the GDMT-withdrawn group, patients who stopped all GDMT classes did not demonstrate a significant difference compared with patients who continued taking a ß-blocker in terms of LVEF (56.7±5.6 GDMT-withdrawn-all-medications vs 55.0±4.0 GDMT withdrawn-but-continued-ß-blocker; p=0.059), LVEDD (50.1±6.3 mm GDMT-withdrawn-all-medications vs 52.1±3.7 mm GDMT withdrawn-but-continued-ß-blocker; p=0.42) or SR maintenance (n=11, 100% GDMT-withdrawn-all-medications vs n=12, 91.7% GDMT withdrawn-but-continued-ß-blocker; p=1.00).

**Table 5:** The number of classes of heart failure medications taken at the baseline and end of follow-up across the whole cohort and the respective groups

Number of classes of GDMT taken by Patients at baseline	Whole cohort (n=88)	GDMT-continued (n=50)	GDMT-withdrawn (n=38)
0 classes, n (%)	0 (0.0)	0 (0.0)	0 (0.0)
1 class, n (%)	5 (5.7)	5 (10.0)	0 (0.0)
2 classes, n (%)	34 (38.6)	19 (38.0)	15 (39.5)
3 classes, n (%)	26 (29.5)	17 (34.0)	9 (23.7)
4 classes, n (%)	23 (26.1)	9 (18.0)	14 (36.8)

Number of classes of GDMT Taken by Patients at the end of follow-up	Whole cohort (n=88)	GDMT-continued (n=50)	GDMT-withdrawn (n=38)
0 classes, n (%)	11 (12.5)	0 (0.0)	11 (28.9)
1 class, n (%)	19 (21.6)	4 (8.0)	15 (39.5)
2 classes, n (%)	30 (34.1)	18 (36.0)	12 (31.6)
3 classes, n (%)	13 (14.8)	13 (26.0)	0 (0.0)
4 classes, n (%)	15 (17.0)	15 (30.0)	0 (0.0)

### Relapse events

One patient in each arm experienced a drop in LVEF below 50% during follow-up (n=1, 2% GDMT-continued vs n=1, 2.6% GDMT-withdrawn; p=1). In each case, this was due to a non-ST elevation myocardial infarction that led to a drop in LVEF.

### Change in LVEF and LVEDD

**Figure 2:**
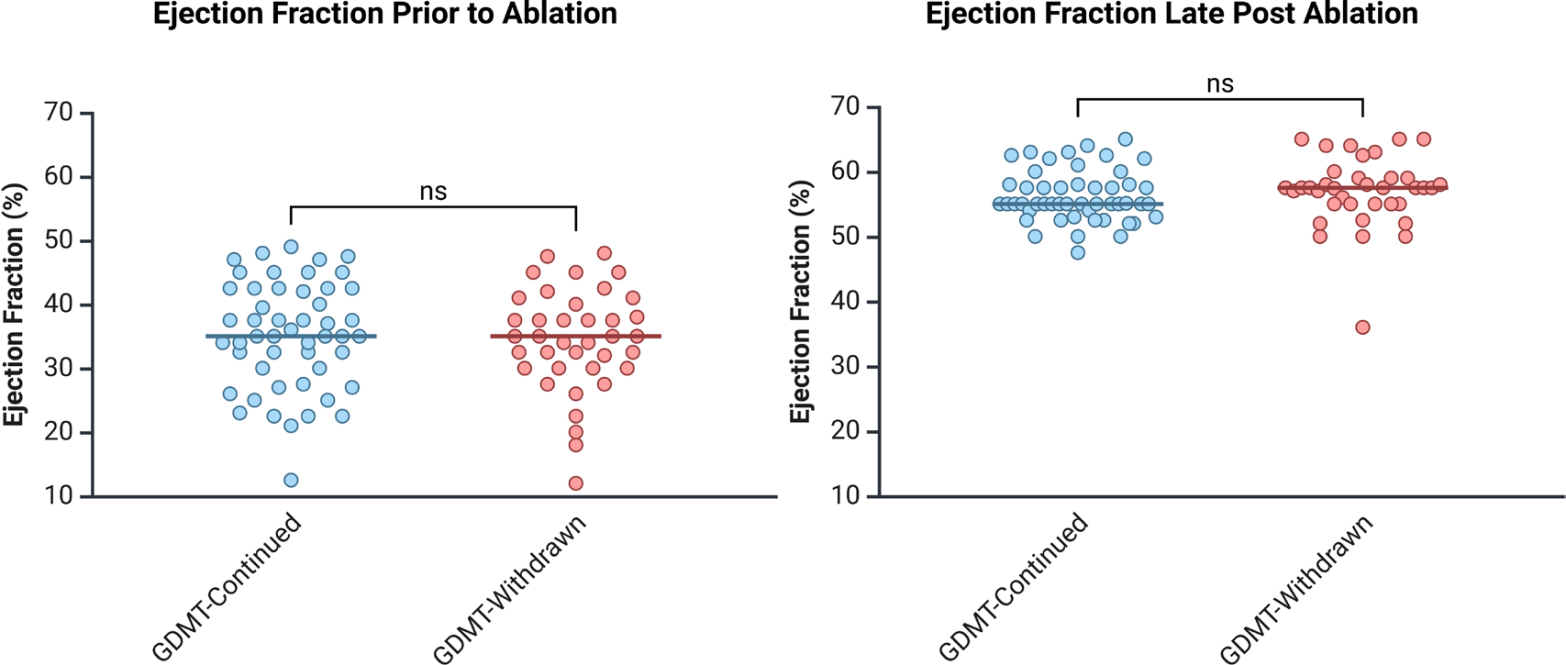
Demonstrates LVEF in the GDMT-continued and GDMT-withdrawn groups prior to catheter ablation (time point A) and at late post catheter ablation (time point C). The figure demonstrates that there is an improvement in LVEF in both groups postablation and that there is no significant difference in LVEF between the GDMT-continued and GDMT-withdrawn group. GDMT, guideline-directed medical therapy; LVEF, left ventricular ejection fraction.

**Figure 3:**
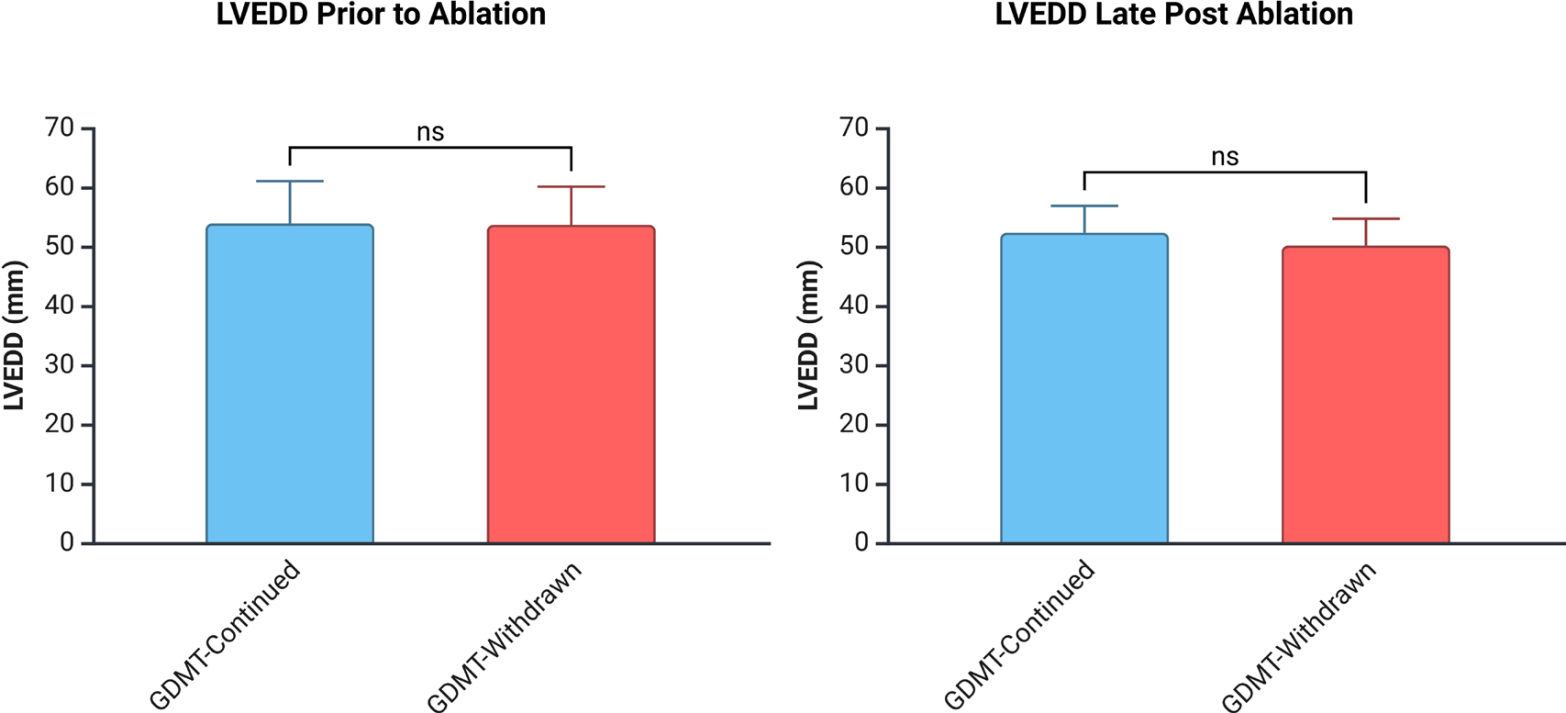
Demonstrates LVEDD in the GDMT-continued and GDMT-withdrawn groups prior to ablation (time point A) and at late post ablation (time point C). The figure demonstrates that there is no significant difference in LVEDD between the GDMT-continued and GDMT-withdrawn group. GDMT, guideline-directed medical therapy; LVEDD, left ventricular end-diastolic diameter.

At the final follow-up, mean LVEF did not differ significantly between groups (56.3±3.8% GDMT-continued vs 56.8±5.5% GDMT-withdrawn; p=0.59) (figure 2). Likewise, the change in LVEF from early postablation (timepoint B) to late follow-up was not significantly different (1.20%±4.32% GDMT-continued vs 0.40%±6.33% GDMT-withdrawn; p=0.48). At the final follow-up, the average LVEDD was also not significantly different between the two groups (49.9±11.9 mm GDMT-continued vs 50.2±4.5 mm GDMT-withdrawn; p=0.89) (figure 3). The change in LVEDD was also not significantly different from early postablation (time point B) to late follow-up (time point C) (0.17±4.88 mm GDMT-continued vs 0.17±4.41 mm GDMT-withdrawn; p=1.00).

In a subgroup analysis of patients who had undergone CMR, there was no difference in LVEF early postablation (timepoint B) between those with LGE and those without (55.1%±3.8% LGE positive vs 55.8%±3.7% LGE negative; p=0.48). In a subgroup analysis excluding patients with LGE on CMR (n=61), mean LVEF at late follow-up remained comparable between groups (56.6%±4.0% GDMT-continued vs 56.0%±5.7% GDMT-withdrawn; p=0.65). In a further subgroup analysis, there was no significant difference in the LVEF at final follow-up (time point C) in patients in the GDMT-withdrawn group who were LGE positive (59.1%±4.2%) compared with those who were LGE negative (56.3±6.7; p=0.26).

### Maintenance of SR

The proportion of patients who remained in SR through final follow-up was high in both groups (41/50, 82.0% GDMT-continued vs 35/38, 92.1% GDMT-withdrawn; p=0.17) and the GDMT-withdrawn group did not have a higher rate of atrial arrhythmia recurrence (9/50, 18% GDMT-continued vs 3/38, 7.9% GDMT-withdrawn; p=0.17). Of those who experienced AF recurrence, 9 (75%) were in persistent AF at their final follow-up and 3 (25%) were in paroxysmal AF.

## Discussion

In this multicentre observational study, patients with AIC and recovered LV systolic function following restoration of SR after catheter ablation who withdrew most of their HF therapies maintained recovered LV systolic function, stable LV dimensions and SR to the same extent as those remaining on GDMT. These findings suggest that in patients with AIC, once AF is durably suppressed and LVEF normalises, lifelong continuation of all GDMT classes may not be universally required.

In this study in the GDMT-withdrawn group, 11/38 (28.9%) patients successfully withdrew all GDMT classes. Importantly, by the time GDMT decisions were made, both groups had equivalent LVEF and LVEDD at timepoint B. This mitigates concerns that patients selected for withdrawal were systematically healthier and reduces the likelihood of selection bias driving the observed outcomes. Those who did not withdraw all classes predominantly remained on one GDMT class, with ß-blockers being the most common GDMT class continued. It is plausible that the ß-blockers were continued to reduce the likelihood of AF recurrence rather than ongoing HF management. It remains unclear whether the continued use of ß-blockade aided in preventing LVSD relapse in the GDMT-withdrawn group. A small observational study showed that 54% (7/13) of patients weaned off metoprolol experienced clinical deterioration (four deaths and three who worsened by NYHA functional class).^21^ Even though small patient numbers, when comparing LVEF, LVEDD and maintenance of SR in those patients who withdrew all GDMT classes with those that only continued ß-blockade, there was no significant difference in these parameters. This potentially highlights that withdrawal of β-blockade remains safe in this patient cohort. However, even though this study has not effectively demonstrated the impact of withdrawal of all GDMT classes in all patients it has highlighted that the withdrawal of a large proportion of these prevents the relapse of LVSD while resulting in a lower pill burden to the patient and lower healthcare costs.

The durability of LV recovery despite GDMT de-escalation carries important clinical implications. If replicated in larger, prospective trials, selective withdrawal could spare patients the lifetime burden of multiple therapies, thereby reducing medication costs, laboratory monitoring, pill burden and the adverse effects of hyperkalaemia, renal impairment, fatigue and orthostatic hypotension. Notably, the Withdrawal of Neurohormal Blockade After Cardiac Resynchronization Therapy (STOP-CRT) trial demonstrated that GDMT discontinuation was safe, only supraventricular tachyarrhythmias and not true systolic dysfunction prompted ß blocker reinitiation.^15^ Likewise, in chemotherapy-induced^22^ and peripartum cardiomyopathy,^23^ HF therapies have been successfully withdrawn once the precipitant resolved without meaningful relapse of LV dysfunction.

To place our results in context, four key studies merit consideration. First, the landmark TRED-HF^16^ trial in non-ischaemic dilated cardiomyopathy enrolled 51 patients with normalised LVEF and withdrew RAAS inhibitors and ß blockers over 6 months. Relapse, defined by ≥10% absolute fall in EF, a >10% increase in LV end-diastolic volume, a doubling of BNP or clinical heart failure, occurred in 44%, prompting guideline recommendations for lifelong therapy in ‘HF with improved EF’. However, TRED-HF's strict inclusion criteria, excluding any patient with LGE on CMR or alternative cardiomyopathy aetiologies, limits its applicability to AF-induced disease. Moreover, 56% of TRED-HF participants maintained recovery despite withdrawal, and long-term follow-up identified a subgroup free of relapse years later, suggesting that GDMT de-escalation may be safe in carefully selected patients even beyond the AIC population.^24^

More recently, Basile *et al* interrogated a Swedish registry of 8728 patients to examine the safety of GDMT withdrawal.^25^ Using a ‘Super Learner’ machine-learning framework, they found that discontinuation of RAAS inhibitors/ARNI or MRAs, but not ß blockers, was linked to higher cardiovascular mortality and heart‐failure hospitalisations. However, their cohort defined LV recovery as EF >40% (not ≥50%), and in the subset with EF ≥50% (n=2979), withdrawal did not affect outcomes. Applying their overall withdrawal rate (~5%) to this subgroup suggests ~150 patients truly de-escalated therapy. Moreover, a substantial proportion of registry subjects had advanced disease with NYHA class III-IV, BNP >1000 pg/mL and severe CKD, sharply contrasting with our postablation AIC population. Thus, while their data caution against GDMT withdrawal in unselected HFrEF patients with EF 40%–50%, their findings may not be generalisable to a carefully phenotyped AIC cohort.

Third, Domínguez-Rodríguez *et al* retrospectively reviewed 200 patients with suspected tachycardia-induced cardiomyopathy, of whom 90 were classified as ‘pure’ arrhythmia-induced.^26^ This excluded patients with ischaemic heart disease, LGE on CMR and significant alcohol use. In this subgroup, withdrawal of RAAS inhibitors (n=6) or β-blockers (n=8) was associated with higher rates of heart-failure hospitalisation or EF decline, with a compounded effect in the single patient in whom both therapies were ceased simultaneously. These outcomes, however, were analysed alongside a larger ‘impure’ cohort, and the authors do not specify how SR and therefore substrate stability were achieved. Indeed, the overall AF recurrence rate was high at 45% over the follow-up period. As Girerd and Jhund note,^27^ clustering of medication changes immediately before clinical deterioration can introduce protopathic bias; clinicians may reduce therapy in response to early symptoms, rather than withdrawal provoking the relapse. Taken together, these factors counsel caution in attributing causality from their findings and may not be applicable to an AIC cohort who have undergone catheter ablation.

Finally, the randomised WITHDRAW-AF trial demonstrated that GDMT cessation after AF ablation was safe in the majority of patients with recovered EF, closely mirroring our real-world results and reinforcing the notion that AIC may be uniquely reversible when durable SR is achieved. Notably, WITHDRAW-AF excluded patients with LGE, whereas our study did not and found similar outcomes between those with and without LGE. This suggests GDMT withdrawal may be feasible even in patients with LGE, although this requires confirmation in larger prospective trials.

The use of oral anticoagulants was significantly less in the GDMT-withdrawn group compared with the GDMT-continued group. It is hard to provide a concrete conclusion to why there was less use of oral anticoagulant drugs despite similar outcomes between the two groups due to the retrospective nature of this study. However, we do speculate that the continuation of GDMT classes potentially results in the patient still being labelled as a HF patient and, as a result, the patient has an ongoing criterion that warrants anticoagulation use. On the contrary, in the withdrawal group, by withdrawing the GDMT classes, it is likely that the label of HF being an underlying comorbidity is disregarded, and thereby so is the indication for anticoagulation. This likely accounts for the difference seen in the use of oral anticoagulant drugs between the two groups.

Our analysis demonstrated that GDMT de-escalation did not compromise maintenance of SR, a finding of particular importance given the link between AF recurrence and rapid LV deterioration in AIC.^28^ Consistent with the WITHDRAW-AF trial, these results suggest that once durable SR is achieved, GDMT withdrawal may be safely undertaken without jeopardising rhythm control.

Mechanistically, AF instigates a self‐reinforcing cycle of electromechanical dyssynchrony, intracellular Ca²^+^ accumulation,^2^ oxidative stress^3^ and elevated filling pressures^29^; substrate insults culminating in adverse LV remodelling.^1^ Ablation‐induced restoration of SR disrupts this cascade and, alongside GDMT, facilitates reverse remodelling. These observations raise the possibility that, once the arrhythmic driver is eliminated and coordinated myocardial mechanics restored, the incremental benefit of chronic neurohormonal blockade may diminish, rendering ongoing GDMT less essential in this setting.

### Limitations

The study’s retrospective, non-randomised design and physician-driven therapy decisions introduce potential selection bias. However, the baseline characteristics were well-matched between the two groups, suggesting no significant selection bias in this study. Stringent imaging requirements improved data validity but reduced the cohort size, as some patients with recovered LVEF would not go on to have final follow-up imaging and were excluded.

Methods of rhythm monitoring differed between patients, which may have led to an underestimation of asymptomatic AF/AT recurrence. However, the primary aim of the study was to evaluate the impact of GDMT withdrawal on LVEF, which was robustly assessed with serial imaging.

Although we defined withdrawal as cessation of ≥50% of GDMT classes, we did not uniformly discontinue all therapies—leaving the possibility that residual medication effects influenced outcomes. However, when comparing those patients who continued β-blocker therapy to those that withdrew all medication, there was no difference in endpoints suggesting that continuation of β-blocker therapy did not influence the outcomes. Not all patients underwent CMR; however, a large proportion did, with no significant difference between the two groups.

The GDMT-withdrawn group had a numerically shorter mean postablation follow-up; however, the difference vs the GDMT-continued group was not statistically significant. Importantly, mean follow-up far exceeded 12 months in both groups, which is longer than typically reported in AF-ablation cohorts and in TRED-HF.

Finally, our sample size is modest. Although data were collected across 12 centres and represent a larger cohort than prior randomised trials, larger, adequately powered randomised studies are required to definitively address the safety of GDMT withdrawal in this population.

## Conclusions

The study findings suggest that lifelong continuation of GDMT may not be universally required for all patients with AIC who achieve sustained recovery of LV systolic function following restoration of SR with catheter ablation. Selective withdrawal, particularly of RAAS inhibitors and MRAs, may be feasible and beneficial in reducing medication burden, laboratory monitoring and adverse effects, thereby improving financial efficiency and patient quality of life. Prospective, randomised trials are warranted to identify which patient subsets can safely forgo GDMT without compromising long-term ventricular performance or arrhythmia outcomes.

## Supplementary material

10.1136/openhrt-2025-003733 online supplemental file 1

## Data Availability

Data are available on reasonable request.
