# Supplementary material for: Withdrawing guideline-directed medical therapy after left ventricular ejection fraction recovery following atrial fibrillation ablation: a multicentre cohort study
Source: Open Heart. 2025 Oct 13;12(2):e003733. doi: 10.1136/openhrt-2025-003733 (PMC12519717; doi:10.1136/openhrt-2025-003733)
Supplement: online supplemental file 1 [file openhrt-12-2-s001.docx]

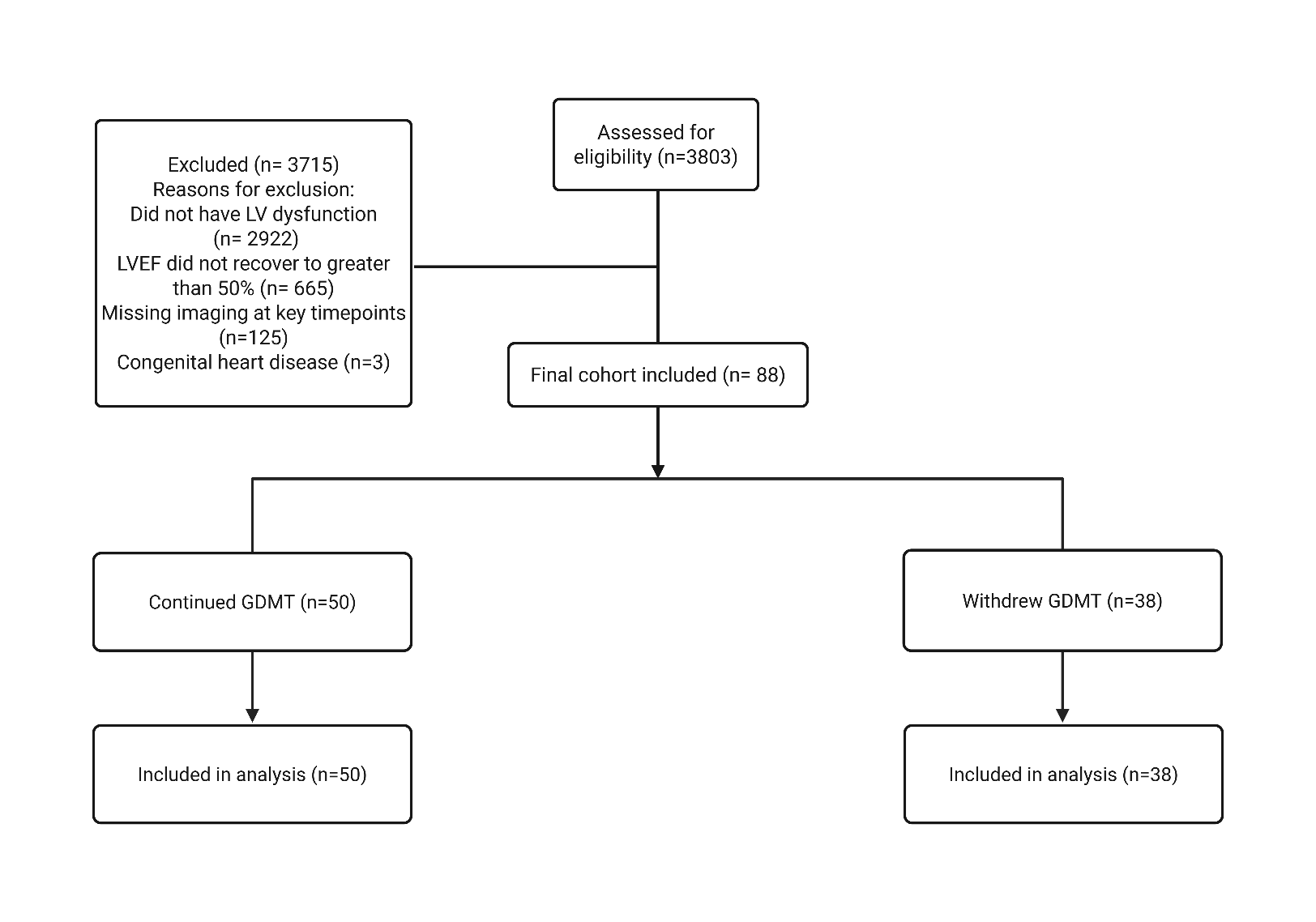


**Supplemental Figure 1-** Demonstrates the Number of Patients Screened, Excluded Due to Predefined Criteria and then the Numbers Included in the Final Analysis Across the GDMT-Continued and GDMT-Withdrawn Groups


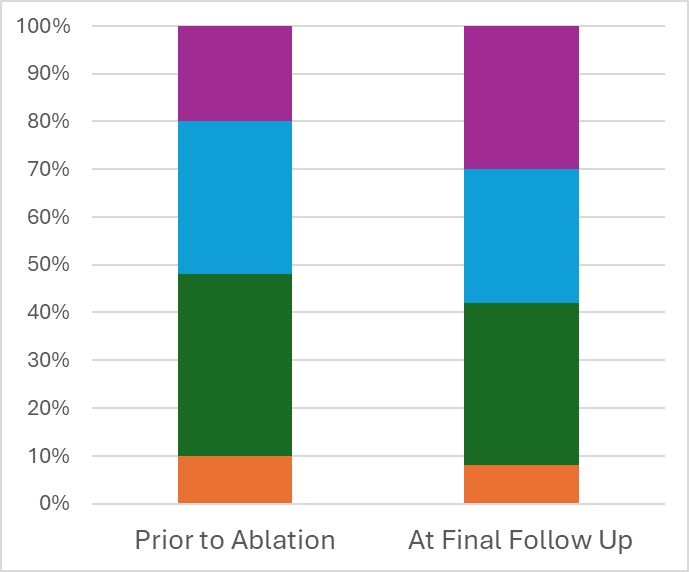

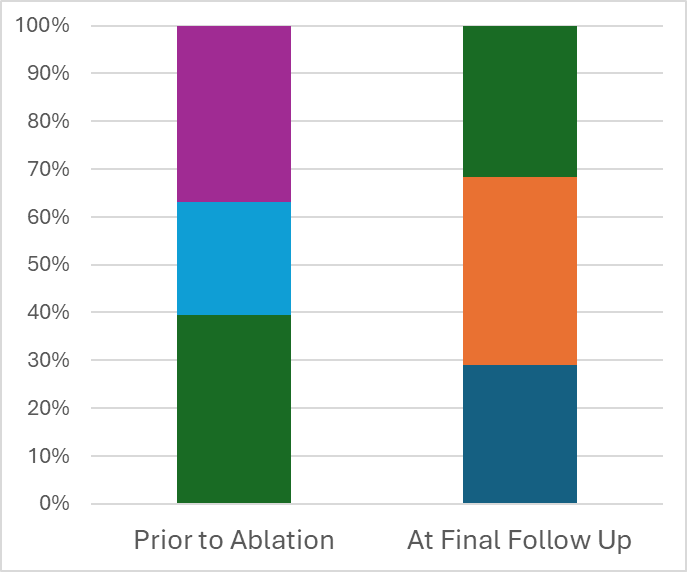

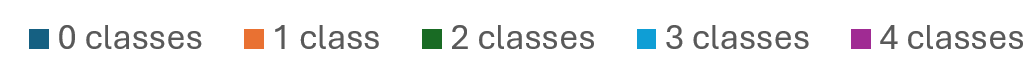


**GDMT-Continued**

**GDMT-Withdrawn**

Percentage of patients

**Supplemental Figure 2** - Demonstrates the GDMT Class Count in the GDMT-Continued and GDMT-Withdrawn groups prior to catheter ablation and late post catheter ablation. The figure demonstrates that post catheter ablation, the patients in the GDMT-Continued group remained on their GDMT classes whilst in the GDMT-Withdrawn group, a large proportion of patients withdraw all their medication with no patients remaining on three or four GDMT classes
